# Evidenzbasierte Arzneimittelinformationen für Kinder und Jugendliche in Deutschland – Sind wir auf dem richtigen Weg?

**DOI:** 10.1007/s00103-026-04239-1

**Published:** 2026-05-08

**Authors:** Ramona Möhrle, Sonja Eberl, Ursula Gramlich, Julia Haering-Zahn, Gabriele Ahne, Tjitske van der Zanden, Saskia de Wildt, Wolfgang Rascher, Antje Neubert

**Affiliations:** 1 Kinder- und Jugendklinik, Friedrich-Alexander-Universität Erlangen-Nürnberg, Loschgestraße 15, 91054 Erlangen, Deutschland; 2 Paediatric and neonatal Intensive Care, Erasmus MC Sophia Children’s Hospital, Rotterdam, Niederlande; 3 Dutch Knowledge Centre Pharmacotherapy for Children, The Hague, Niederlande; 4 Departments of Pharmacy, Pharmacology and Toxicology, Radboud University Medical Centre Nijmegen, Nijmegen, Niederlande

**Keywords:** Evidenzbasierte Arzneimittelinformation, Arzneimitteltherapiesicherheit, Pädiatrie, Nutzerumfrage, Arzneimitteldatenbank, Evidence-based drug information, Drug therapy safety, Paediatrics, User survey, Drug database

## Abstract

**Einleitung:**

Kinder und Jugendliche sind bei der Arzneimitteltherapie benachteiligt, da Medikamente in dieser Altersgruppe häufig außerhalb der Zulassung (off-label) angewendet werden. Trotz der Zunahme an Kinderzulassungen durch die EU-Kinderarzneimittelverordnung werden Off-Label-Anwendungen weiterhin Teil der pädiatrischen Therapien bleiben. Die Online-Plattform „Kinderformularium.DE“ stellt seit 2021 evidenzbasierte Dosierungsempfehlungen für den On- und Off-Label-Bereich in Deutschland bereit und hat sich als feste Informationsquelle in der pädiatrischen Praxis etabliert. Ziel dieser Arbeit ist es, den Umfang und den Zulassungsstatus der enthaltenen altersspezifischen (Off-Label‑)Dosierungsempfehlungen zu analysieren und die Nutzung und Akzeptanz zu evaluieren.

**Methoden:**

In den Wirkstoffmonographien wurden die Verfügbarkeit und der Zulassungsstatus altersspezifischer Dosierungsempfehlungen unter Berücksichtigung unterschiedlicher Indikationen und Applikationswege untersucht. Eine Nutzerumfrage (von 11/2024 bis 02/2025) wurde mittels Mixed-Methods-Analyse ausgewertet.

**Ergebnisse:**

Kinderformularium.DE enthält 637 Monographien mit 5536 spezifischen Dosierungsempfehlungen. Diese sind altersabhängig verteilt: Für Früh- und Neugeborene gibt es weniger Dosierungsinformationen, die zudem häufiger als off-label eingestuft sind. Die hohe Akzeptanz der Plattform, die vor allem von Ärztinnen und Ärzten häufig genutzt wird, bestätigt ihren Stellenwert in der pädiatrischen Versorgung.

**Diskussion:**

Mit seinen altersspezifischen (Off-Label‑)Dosierungsempfehlungen ist Kinderformularium.DE ein wichtiges Instrument zur Förderung der Arzneimitteltherapiesicherheit in der pädiatrischen Versorgung in Deutschland. Die Plattform muss weiterhin kontinuierlich aktualisiert und ergänzt werden.

**Zusatzmaterial online:**

Zusätzliche Informationen sind in der Online-Version dieses Artikels (10.1007/s00103-026-04239-1) enthalten.

## Abstract (en)

**Introduction:**

Children and adolescents are disadvantaged when it comes to drug therapy, as drugs are frequently used off-label for this age group. Despite regulatory measures such as the EU Paediatric Regulation, off-label use in paediatrics remains widespread. Since 2021, the online platform “Kinderformularium.DE” has been providing evidence-based dosing recommendations for on- and off-label drug use in Germany, establishing itself as reliable source of information in paediatric practice. This study aims to analyse the scope of age-appropriate (off-label) dosage recommendations provided and to evaluate usage patterns and user satisfaction.

**Methods:**

Drug monographs were examined to determine the availability and licensing status of age-specific dosing recommendations, taking into account different indications and routes of administration. A user survey was conducted between November 2024 and February 2025 and analysed using a mixed-methods approach.

**Results:**

Kinderformularium.DE contains 637 monographs with 5536 individual age-specific dosage recommendations. Both the number of dosage recommendations and the licensing status vary by age: there is less dosage information available for preterm and newborn infants, and this information is more frequently classified as off-label. The survey confirms the platform’s high level of acceptance—it is used frequently, particularly by doctors—and highlights its importance in the care of paediatric patients.

**Discussion:**

With its age-specific (off-label) dosage recommendations, Kinderformularium.DE is an important tool for ensuring medication safety in paediatric clinical practice in Germany. The platform must continue to be updated and expanded on an ongoing basis.

## Einleitung

Die Arzneimitteltherapie in der Kinder- und Jugendmedizin ist aufgrund der häufigen Therapien außerhalb der Zulassung, nichtvalidierter Dosisempfehlungen und fehlender pädiatrischer Darreichungsformen problematisch und anfällig für Medikationsfehler [1–5]. Mit der Einführung der Kinderarzneimittelverordnung (EG Nr. 1901/2006) im Jahr 2007 wurden verschiedene Maßnahmen implementiert, welche die pädiatrische Arzneimitteltherapie sicherer und die Entwicklung von Arzneimitteln für Kinder vorantreiben sollten. Die Zahl der klinischen Studien mit Kindern ist dadurch gestiegen und hat vor allem zur Zulassung von innovativen Medikamenten bei Kindern geführt. Jedoch sind ältere, lang etablierte Medikamente mit abgelaufenem Patentschutz (Generika), die trotz fehlender pädiatrischer Zulassung häufig in der Kinder- und Jugendmedizin off-label eingesetzt werden, aus wirtschaftlichen Gründen von der Forschung durch pharmazeutische Unternehmen weitestgehend ausgenommen [6, 7]. Auch der speziell hierfür geschaffene finanzielle Anreiz in Form einer *Paediatric Use Marketing Authorisation *(PUMA) nach Artikel 30 der Kinderarzneimittelverordnung aus dem Jahr 2007 hat bisher lediglich zu 8 Zulassungen geführt. Das Ergebnis dieses speziell für Kinder eingerichteten Zulassungsverfahrens wurde von der Europäischen Kommission als Enttäuschung bewertet [8].

Aus diesem Grund werden evidenzbasierte Daten aus Zulassungsstudien für Generika zur korrekten und sicheren Anwendung bei pädiatrischen Patientengruppen auch zukünftig häufig fehlen, sodass viele Therapien weiterhin im Rahmen einer Off-Label-Anwendung erfolgen. Diese ist mit Risiken verbunden und anfällig für fehlerhafte oder suboptimale Dosierungen. Verschreibende tragen eine besondere Verantwortung, die verfügbare Evidenz zu berücksichtigen, um altersentsprechende Dosierungen auszuwählen sowie Nutzen und Risiko sorgfältig abzuwägen [9]. Hierfür benötigen sie Zugang zu verlässlichen und aktuellen Informationen für alle relevanten Arzneimittel.

Vor 2021 gab es in Deutschland weder eine Datenbank noch ein Standardwerk, in dem systematisch und auf klinisch-pharmakologischer Evidenz basierend der wissenschaftliche Stand zur Anwendung von Medikamenten bei Kindern und Jugendlichen zu finden war. Die meisten vorhandenen Informationsquellen, unter anderem die sogenannten Kitteltaschenbücher, enthielten empirisch gesammelte Informationen ohne Angabe der Informationsquelle oder des Evidenzgrades. In anderen Ländern, wie zum Beispiel in Großbritannien, den Niederlanden und der Schweiz, gab es bereits durch staatlich geförderte nationale Datenbanken für Kinderarzneimittel einen signifikanten Fortschritt in der pädiatrischen Pharmakotherapie [10–13].

Im Rahmen des Aktionsplans zur Verbesserung der Arzneimitteltherapiesicherheit (AMTS) des Bundesministeriums für Gesundheit wurde an der Kinder- und Jugendklinik Erlangen seit 2014 eine Arzneimittelinformationsplattform für Kinder und Jugendliche entwickelt. Seit 2017 basiert sie auf einer Lizenzvereinbarung mit dem niederländischen Best-Practice-Modell „Kinderformularium.NL“ [13–15]. Mit den Niederlanden und weiteren Ländern (Österreich und Norwegen im Rahmen ähnlicher Lizenzvereinbarungen) hat sich in den letzten Jahren eine dauerhafte enge Zusammenarbeit entwickelt mit dem Ziel, die pädiatrische Arzneimitteltherapie auf europäischer Ebene zu harmonisieren und den Verschreibenden die Möglichkeit zu geben, Kinder mit einer auf der besten Evidenz basierenden Arzneimitteltherapie zu versorgen.

Der Nutzen von Kinderformularium.DE wurde im Vorfeld der Freischaltung für alle Angehörigen der Gesundheitsberufe zwischen Oktober 2018 und Juni 2020 zusammen mit weiteren Maßnahmen wie pädiatrisch-pharmakologischen Qualitätszirkeln und der Meldung von unerwünschten Arzneimittelwirkungen und Medikationsfehlern in der pädiatrischen Praxis durch eine Förderung des Innovationfonds des Gemeinsamen Bundesausschusses evaluiert. Es konnte ein positiver Effekt auf die AMTS gezeigt werden [16].

Die Freischaltung der kostenfreien Datenbank in Deutschland erfolgte 2021. Seitdem wurde Kinderformularium.DE in Zusammenarbeit mit den Niederlanden kontinuierlich weiterentwickelt und um neue Monographien bzw. Einträge ergänzt. Neben Informationen zu speziell für Kinder zugelassenen Arzneimitteln umfasst sie auch Wirkstoffe, für die in der wissenschaftlichen Literatur ein positives Nutzen-Risiko-Verhältnis beschrieben ist und die als sachgerechte Off-Label-Anwendungen gelten. Die Datenbank enthält somit neben Angaben aus den Fachinformationen (On-Label-Use) auch evidenzbasierte Informationen aus der klinischen Praxis (Off-Label-Use) und gibt differenzierte Empfehlungen für verschiedene Indikationen und Altersgruppen. Auch seltene Erkrankungen werden berücksichtigt. Eine Untersuchung der Verordnungen für Patientinnen und Patienten einer allgemeinpädiatrischen Station aus den Jahren 2014 bis 2019 hat gezeigt, dass für 93 % dieser Verordnungen Informationen im Kinderformularium.DE hinterlegt waren [17].

Die vorliegende Arbeit hat zum Ziel, die Inhalte von Kinderformularium.DE, insbesondere die Verfügbarkeit und den Zulassungsstatus von Dosierungsinformationen für die verschiedenen Altersgruppen, zu beschreiben sowie die Akzeptanz der Datenbank durch die Nutzenden zu evaluieren. Die Ergebnisse der Analyse und der Nutzerumfrage dienen dazu, die Bedeutung der Plattform in der Praxis zu ermitteln und eine Grundlage für künftige Optimierungen zu schaffen.

## Methoden

### Analyse des Kinderformularium.DE

Die Datenbank Kinderformularium.DE ist in wirkstoffbasierte Monographien gegliedert. Diese sind unter Berücksichtigung der relevanten Indikationen und Applikationswege in Abschnitte unterteilt. Jeder Abschnitt enthält für verschiedene Altersgruppen spezifische Dosierungsempfehlungen. Das heißt, eine altersspezifische Dosierungsempfehlung (Datensatz) bezieht sich auf die Kombination eines Wirkstoffs, einer Indikation, eines Applikationswegs und einer spezifischen pädiatrischen Altersgruppe.

Angelehnt an die Klassifikation des International Council for Harmonisation of Technical Requirements for Pharmaceuticals for Human Use (ICH) wurden bei der Auswertung 6 Altersgruppen definiert: Frühgeborene (Gestationsalter < 37 Wochen), Neugeborene (0 bis < 28 Tage), Säuglinge/Kleinkinder (28 Tage bis < 2 Jahre), Vorschulkinder (2 bis < 6 Jahre), Schulkinder (6 bis < 12 Jahre) und Jugendliche (12 bis < 18 Jahre).

Jeder Datensatz wurde basierend auf den Angaben im Kinderformularium.DE als on- oder off-label gekennzeichnet. Die Vorgehensweise zur Einstufung des Zulassungsstatus in off-label und on-label ist im Kinderformularium.DE unter „Nutzungshinweise“ dargestellt. Infobox 1 gibt einen Überblick über die angewendeten Kriterien zur Bewertung des Zulassungsstatus. Sofern sich innerhalb einer Altersklasse der Zulassungsstatus änderte, wurde der betroffene Datensatz auch dann als on-label bezeichnet, wenn dies nur für einen Teil der entsprechenden Altersklasse zutraf. So wurde bei einem Wirkstoff mit einer Zulassung ab 3 Jahren die Dosierungsempfehlung für die Altersgruppe der Vorschulkinder (2 bis < 6 Jahre) entsprechend als on-label eingestuft.

Zur Charakterisierung des Datenbankumfangs wurde in jedem Abschnitt die Verfügbarkeit von (Off-Label‑)Dosierungsinformationen für die 6 definierten Altersklassen geprüft. Anschließend wurde für jede Altersgruppe der Anteil an Abschnitten mit Empfehlung ermittelt. Darüber hinaus wurde der Prozentsatz der Off-Label-Dosierungsempfehlungen pro Altersgruppe bestimmt.

#### Infobox 1 Kriterien für die Beurteilung des Zulassungsstatus im Kinderformularium.DE

Im Kinderformularium.DE gelten alle Arzneimittelanwendungen als off-label, die nicht durch die Angaben in der jeweiligen Fachinformation abgedeckt sind (on-label).

Die Prüfung erfolgt in Bezug auf:

- Indikation,
- Applikationsweg,
- Alter,
- Dosis.

Wenn verschiedene Fertigarzneimittel für einen Wirkstoff zugelassen sind, wird im Kinderformularium.DE die „breiteste“ Zulassung angegeben.

### Nutzerumfrage

Die Nutzerumfrage umfasste insgesamt 18 Fragen, mit denen die folgenden 3 Aspekte erfasst wurden: 1) Nutzercharakteristika, 2) die Art und Weise der Nutzung und 3) die Bewertung der Zufriedenheit mit der Datenbank(struktur). Der Fragebogen orientierte sich weitgehend an einer Umfrage aus dem Jahr 2022, um einen Vergleich der Ergebnisse beider Umfragen zu ermöglichen [18]. Die meisten Fragen wurden im Single- oder Multiple-Choice-Format gestellt. In einigen Fällen war auch eine Freitextantwort möglich. Um den Grad der Zustimmung zu Aussagen über Kinderformularium.DE zu erfassen, wurde eine 5‑Punkte-Likert-Skala verwendet (siehe Fragebogen im Onlinematerial).

Die Umfrage wurde anonym mit dem Tool SoSci Survey (https://www.soscisurvey.de/) durchgeführt. Der Fragebogen war von November 2024 bis Februar 2025 auf der Website www.kinderformularium.de zugänglich. Um eine ausreichende Anzahl von Teilnehmenden zu erreichen, wurde auf der Website ein Pop-up-Fenster und ein Informationstext auf der Startseite eingeblendet. Darüber hinaus wurde die Umfrage in den sozialen Medien (LinkedIn, Instagram) sowie im Newsletter von Kinderformularium.DE beworben.

Zur Auswertung der Antworten wurde ein Mixed-Methods-Ansatz verwendet, das heißt, sowohl quantitative als auch qualitative Methoden wurden genutzt: Bei den Single- und Multiple-Choice-Fragen wurde jeweils der Prozentsatz bestimmt, zu dem die einzelnen Antworten ausgewählt wurden. Darüber hinaus erfolgte eine Berechnung der Häufigkeitsverteilungen der Antworten auf die Likert-Skala-Fragen (verwendete Software: SPSS, Version 29.0.1.0). In einer qualitativen Auswertung wurden die Freitextantworten einer deskriptiven Auswertung unterzogen.

## Ergebnisse

### Verfügbarkeit von altersspezifischen (Off-Label‑)Dosierungsempfehlungen im Kinderformularium.DE

**Abb. 1:**
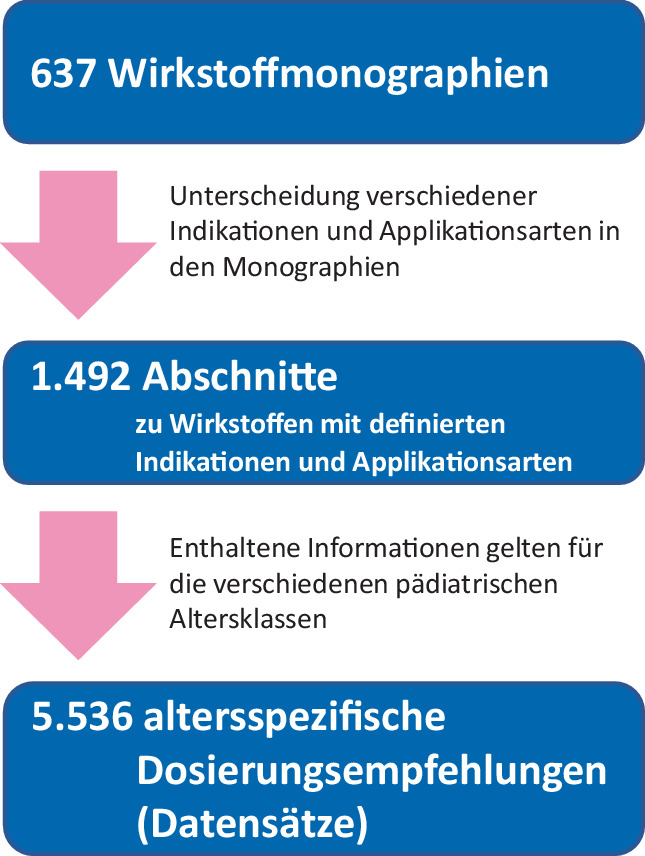
Analyse von Kinderformularium.DE – Unterteilung der Wirkstoffmonographien in Abschnitte und einzelne Datensätze mit altersspezifischen Dosierungsempfehlungen

Stand Februar 2024 umfasste die Datenbank Kinderformularium.DE 637 Monographien. Bei der Auswertung wurden die verschiedenen Indikationen und Applikationswege berücksichtigt, wodurch sich 1492 Abschnitte ergaben. Diese enthielten insgesamt 5536 individuelle altersabhängige Dosierungsempfehlungen (Datensätze; Abb. 1).

Von den 637 Monographien enthielten 98 % (626) mindestens eine Dosierungsinformation für Jugendliche, 94 % (600) für Schulkinder, 85 % (538) für Vorschulkinder, 72 % (458) für Säuglinge und Kleinkinder, 36 % (226) für Neugeborene und 12 % (73) für Frühgeborene.

**Abb. 2:**
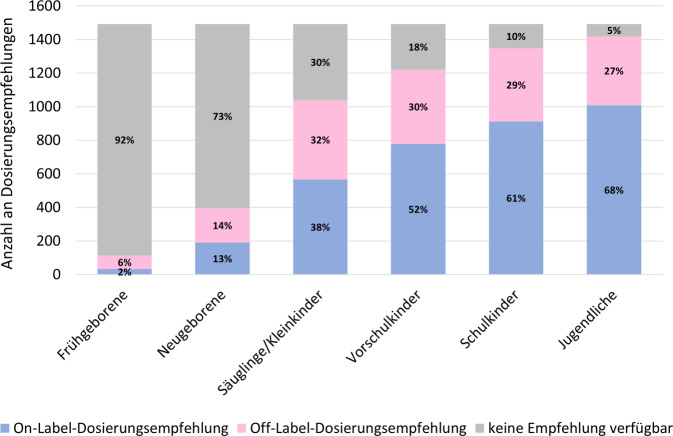
Verteilung der Dosierungsempfehlungen in den verschiedenen Altersgruppen innerhalb der 1492 Abschnitte der Monographien

In Abb. 2 ist die Verteilung der Dosierungsempfehlungen auf die verschiedenen Altersgruppen dargestellt. Es zeigte sich eine größere Verfügbarkeit an Dosierungsempfehlungen mit zunehmendem Alter: Für die Gruppe der Frühgeborenen war in 8 % (114) der insgesamt 1492 Abschnitte eine Empfehlung verfügbar, für die Gruppe der Neugeborenen in 27 % (395) der Abschnitte. Für die Altersgruppen der Säuglinge/Kleinkinder bis Jugendlichen waren dagegen in 70–95 % (1039–1419) der Abschnitte eine Dosierungsempfehlung enthalten.

**Abb. 3:**
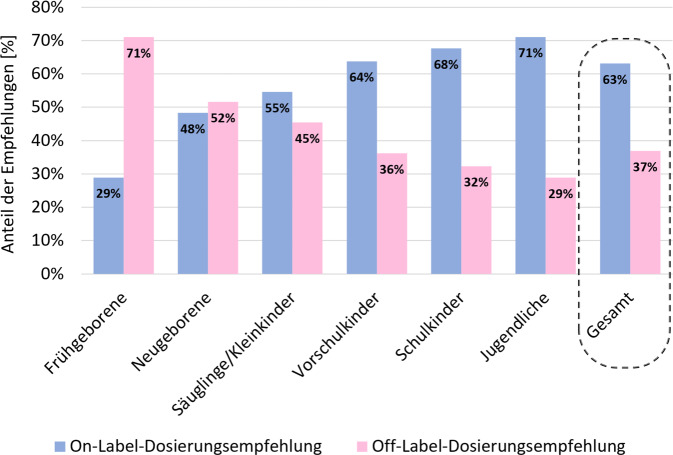
Anteile der Off-Label- und On-Label-Dosierungsempfehlungen in den verschiedenen Altersgruppen basierend auf insgesamt 5536 Empfehlungen

Von den insgesamt 5536 Dosierungsempfehlungen wurden 37 % (2045) als off-label und 63 % (3491) als on-label eingestuft (Abb. 3). Der Zulassungsstatus war dabei stark altersabhängig: Der Anteil der Off-Label-Dosierungen sank mit zunehmendem Alter. 71 % (81/114) der Dosierungsempfehlungen für Frühgeborene und 52 % (204/395) der Empfehlungen für Neugeborene waren off-label. Bei Schulkindern und Jugendlichen war das nur für 32 % (436/1348) bzw. 29 % (410/1419) der Fall.

### Bedeutung und Weiterentwicklungsmöglichkeiten von Kinderformularium.DE

#### Charakterisierung der Nutzenden.

651 Personen nahmen an der Umfrage teil. Bei der Mehrheit der Befragten (95 %) handelt es sich um Angehörige medizinischer oder pharmazeutischer Berufsgruppen, nur eine geringe Anzahl der Befragten gab einen anderen beruflichen Hintergrund an. Insgesamt 74 % der Befragten sind im stationären Bereich tätig. Die Nutzenden stammen aus allen abgefragten Altersgruppen, wodurch ein breites Spektrum an beruflicher Erfahrung abgedeckt wird (Tab. 1).

**Tab. 1:** Charakterisierung der Datenbanknutzenden (*N* = 651)

Merkmal	Ausprägung	Anteil (Anzahl *n*)
Beruf	Ärztinnen/Ärzte	71 % (461)
	Pflegepersonal	1 % (8)
	Apothekerinnen/Apotheker	22 % (146)
	Pharmazeutische Assistentinnen/Assistenten	1 % (7)
	Studierende	1 % (6)
	Betreuende	2 % (11)
	Andere	2 % (12)
Arbeitsplatz	Stationäre Versorgung	62 % (406)
	Ambulante Versorgung	16 % (105)
	Krankenhausapotheke	12 % (75)
	Öffentliche Apotheke	9 % (58)
	Keine Angabe	1 % (7)
Alter	18–25 Jahre	2 % (12)
	26–35 Jahre	36 % (235)
	36–45 Jahre	29 % (188)
	46–55 Jahre	21 % (137)
	56–65 Jahre	11 % (70)
	66–75 Jahre	1 % (6)
	≥ 76 Jahre	0 % (2)
	Keine Angabe	0 % (1)

#### Datenbanknutzung.

Fast die Hälfte der Befragten (46 %) gab an, die Datenbank bereits seit mehreren Jahren zu nutzen, 31 % nutzen sie seit einem Jahr. Die Datenbank wird von 77 % der Befragten mehrmals täglich bis einmal wöchentlich konsultiert.

**Abb. 4:**
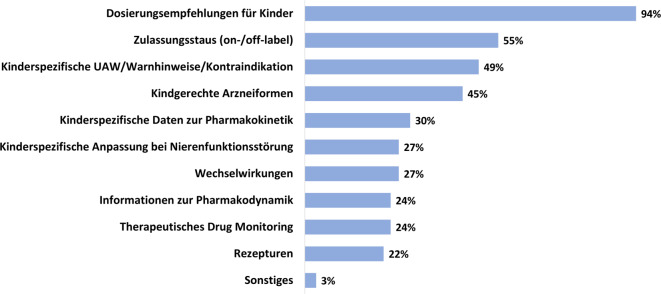
Anteile der gesuchten Informationen im Kinderformularium.DE. *UAW:* *unerwünschte Arzneimittelwirkungen*

Der überwiegende Teil der 651 befragten Nutzenden sucht Informationen zu Dosierungsempfehlungen (94 %; 613). Der Zulassungsstatus (55 %; 358), kinderspezifische Nebenwirkungen (einschließlich Warnhinweise und Kontraindikationen; 49 %; 322) und geeignete Darreichungsformen für Kinder (45 %; 292) sind ebenfalls häufig gesuchte Informationen genauso wie kinderspezifische pharmakokinetische Daten (30 %; 195) und Dosisanpassungen bei Nierenfunktionsstörungen (27 %; 174) (Abb. 4).

16 % bzw. 74 % der Befragten befolgen die Dosierungsempfehlungen immer oder meistens. 4 % befolgen sie manchmal und 6 % machten keine Angaben.

Neben den Monographien werden auf der Webseite unter den „Zusatzinformationen“ auch ergänzende Informationen, beispielsweise zu problematischen Hilfsstoffen, zu bestimmten Erkrankungen oder zu geeigneten Darreichungsformen, bereitgestellt. 35 % der Befragten nutzen diese Zusatzinformationen regelmäßig, 47 % manchmal und 2 % nie. Der Rest der Befragten hat keine Kenntnis davon oder machte keine Angabe (16 %).

19 % der Befragten gaben an, stets eine Antwort auf ihre Frage im Kinderformularium.DE zu finden, bei 77 % ist das häufig der Fall, 4 % machten keine Angabe.

#### Zufriedenheit mit der Datenbank(struktur).

**Abb. 5:**
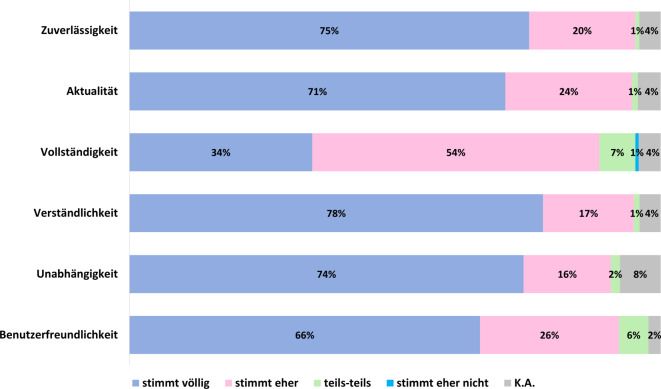
Zufriedenheit mit Kinderformularium.DE – Zustimmung zu den einzelnen Kriterien. *K.A:* *keine Angabe*

Die Zufriedenheit mit Kinderformularium.DE wurde anhand der Kriterien Zuverlässigkeit, Aktualität, Vollständigkeit, Verständlichkeit, Unabhängigkeit und Benutzerfreundlichkeit evaluiert. Mehr als 90 % der Befragten stimmten den genannten Attributen entweder vollständig oder teilweise zu. Die Verständlichkeit der dargestellten Informationen wurde mit der höchsten Zustimmung bewertet: 78 % der Befragten stimmten dieser Aussage voll und ganz zu, 17 % stimmten ihr teilweise zu (Abb. 5).

Zum Abschluss der Befragung wurde den Teilnehmenden die Gelegenheit gegeben, eine abschließende Rückmeldung in einem Freitextfeld zu verfassen. Es wurden insgesamt 163 Antworten in Form von Kommentaren und Vorschlägen verfasst. Die am häufigsten genannte Anregung zur Weiterentwicklung des Kinderformularium.DE war die Einführung einer App (17 %; 28). Außerdem wurden einige weitere technische (12 %; 19) und inhaltliche (17 %; 28) Verbesserungen vorgeschlagen. Beispielsweise wurde der Wunsch nach Informationen zur Kompatibilität mit Trägerlösungen oder anderen Medikamenten bei intravenöser Applikation geäußert. Zudem wurde darauf hingewiesen, dass bestimmte Wirkstoffe im Kinderformularium.DE nicht enthalten sind. Teilweise blieb den Nutzenden unklar, ob das Fehlen auf die eingeschränkte Eignung des Arzneimittels in der Pädiatrie zurückzuführen ist oder der Wirkstoff noch als Monographie ergänzt werden sollte. Als Beispiele für zu ergänzende Wirkstoffe wurden unter anderem Apixaban, Ivabradin und Magnesiumsulfat genannt. Schließlich lobten 44 % (71/163) der Freitextantworten das Kinderformularium.DE.

## Diskussion

Kinderformularium.DE stellt seit Januar 2021 evidenzbasierte Informationen zur Dosierung und Anwendung von Arzneimitteln bei Kindern und Jugendlichen für zugelassene und Off-Label-Anwendungen bereit. Die Anzahl der verfügbaren Monographien hat sich seit der Freischaltung im Jahr 2021 fast verdoppelt (2021 *n* = 387 vs. 2024 *n* = 637; [18]). Zum Zeitpunkt der Analyse standen 5536 altersentsprechende Dosierungsinformationen zur Verfügung. Die Ergebnisse der Nutzerumfrage bestätigen die hohe Akzeptanz der Plattform in Deutschland und ihren Stellenwert bei der Versorgung pädiatrischer Patientinnen und Patienten.

Die Analyse zeigt, dass die verfügbaren Informationen stark altersabhängig sind. Nur ein Drittel (36 %) der Monographien enthält Empfehlungen für Neugeborene, für Frühgeborene ist es ein Zehntel (12 %). Berücksichtigt man neben dem Wirkstoff auch die Indikation und den Applikationsweg, so stehen nur in 27 % der Abschnitte Dosierungsempfehlungen für Neugeborene und in 8 % dieser Abschnitte Dosierungsempfehlungen für Frühgeborene zur Verfügung.

Dies spiegelt zum einen das kleinere Therapiespektrum in diesen beiden Altersgruppen wider, bestätigt aber auch, dass hier sehr viel weniger Erfahrung und Evidenz hinsichtlich der Arzneimitteltherapien vorliegt [19, 20]. Die Herausforderungen bei der Durchführung von klinischen Studien in der Neonatologie sind vielfältig. Der sich schnell entwickelnde Stoffwechsel, große interindividuelle Variabilität und die ethischen Anforderungen an Studien mit dieser Patientenpopulation sind nur einige Gründe für den hier noch immer bestehenden Mangel an evidenzbasierten Arzneimitteltherapien [21]. Die ins Leben gerufene „Lancet Child and Adolescent Health Commission“, die sich mit der Zukunft der Neonatologie befasst, spricht in einer aktuellen Veröffentlichung von einem globalen Public-Health-Problem und weist auf den dringenden Handlungsbedarf hin [20].

Auch für andere Altersgruppen enthalten die Monographien nicht immer passende, altersentsprechende Empfehlungen, wenn auch in deutlich geringerem Umfang als bei den Früh- und Neugeborenen. Dies lässt sich damit begründen, dass nicht alle Therapien für alle Altersgruppen relevant sind, wie beispielsweise die Behandlung von neonatalen Apnoen mit Koffein oder die Prophylaxe eines Vitamin-K-Mangels nach der Geburt [22]. Untersuchungen von Verordnungsdaten sind notwendig, um herauszufinden, in welchem Umfang die Empfehlungen in Kinderformularium.DE tatsächlich die in der Praxis eingesetzten Therapien abdecken, und um Lücken zu identifizieren.

Von den 5536 altersentsprechenden Dosierungsempfehlungen ist circa ein Drittel (37 %) als off-label eingestuft. Dieses Ergebnis ist vergleichbar mit Studien, in welchen über alle pädiatrischen Altersgruppen circa 30 % der Verordnungen als off-label eingestuft werden [23]. Wimmer et al. haben deutsche Fachinformationen hinsichtlich der Verfügbarkeit von pädiatrischen Informationen untersucht und gezeigt, dass 40 % aller Fachinformationen mindestens einen Warnhinweis oder eine Gegenanzeige betreffend der Anwendung im Kindesalter enthalten [24].

Van der Zanden et al. untersuchten das Dutch Paediatric Formulary (DPF) und fanden, dass 42 % der altersentsprechenden Dosierungsempfehlungen in den Niederlanden off-label sind. DPF ist inhaltlich die Grundlage für Kinderformularium.DE, wobei der Zulassungsstatus individuell für jedes Land ermittelt wird [25]. Die Unterschiede zwischen diesen beiden Auswertungen bestätigen die länderspezifischen Besonderheiten bei den Zulassungen und die Notwendigkeit, Informationen wie den Zulassungsstatus auf nationaler Ebene zu betrachten.

Für eine optimale Therapie mit einem minimalen Risiko für die Patientinnen und Patienten hat der Zulassungsstatus nicht die größte Aussagekraft. Rechtlich gesehen wird eine Zulassung nur dann erteilt, wenn der Hersteller die Qualität, Wirksamkeit und Unbedenklichkeit nachgewiesen und die Zulassung beantragt hat. Das war in der Vergangenheit nicht immer so, sodass es heute noch „Altzulassungen“ für bestimmte Altersgruppen und Indikationen gibt, für die keine kontrollierten Studien vorliegen. Auf der anderen Seite sollten Off-Label-Therapien durch sehr gute wissenschaftliche Evidenz belegt sein [25]. Die Untersuchung von van der Zanden et al. hat gezeigt, dass nur 14 % der Off-Label-Dosierungsempfehlungen Metaanalysen oder randomisierte kontrollierte Studien zugrunde liegen [25].

Für pharmazeutische Unternehmen ist es wirtschaftlich nicht attraktiv, randomisiert-kontrollierte Studien für Arzneimittel ohne Patentschutz durchzuführen, um fehlende Daten für bestimmte Indikationen und Altersgruppen zu sammeln, die das Evidenzniveau verbessern würden. Zur Umsetzung solcher Studien wären staatliche Fördermittel oder Gelder aus Non-Profit-Organisationen wünschenswert. Darüber hinaus ist es wichtig, Informationslücken durch die gezielte Erhebung von fehlenden Pharmakokinetik‑, Sicherheits- und/oder Wirksamkeitsdaten oder durch Extrapolation zu schließen. Die Verfügbarkeit der besten verfügbaren Evidenz als Grundlage für die Anwendung von Arzneimitteln im Off-Label-Bereich ist ein weiterer wichtiger Ansatz, um Therapien zu standardisieren, zu optimieren und die Patientensicherheit zu verbessern.

Die aktuelle Nutzerumfrage zeigt eine große Übereinstimmung mit den Ergebnissen der Umfrage von 2022, was auf eine Kontinuität im Nutzungsverhalten und der Zufriedenheit schließen lässt [18].

Der Anteil an täglichen und wöchentlichen Nutzenden ist erheblich gestiegen, was sich auf den gestiegenen Bekanntheitsgrad sowie den Übergang in die pädiatrische Praxis zurückführen lässt. Im Vergleich zur vorherigen Umfrage zeigte sich eine Zunahme der Nutzung von Kinderformularium.DE unter Apothekerinnen und Apothekern, insbesondere in Krankenhausapotheken. Wie bereits in der 2022 durchgeführten Umfrage gehört auch dieses Mal der überwiegende Teil der Nutzenden (72 %) zum medizinischen Fachpersonal. Das heißt vor allem Ärztinnen und Ärzte benutzen die Plattform für ihre tägliche Arbeit. Der Fokus von Kinderformularium.DE auf der Bereitstellung von Off-Label-Dosierungsempfehlungen macht sie zu einer für diese Berufsgruppe besonders wertvollen Informationsquelle. Bei der Verordnung von Off-Label-Medikamenten können sich die Ärztinnen und Ärzte nicht an den Informationen in der Fachinformation orientieren, sondern müssen unter Berücksichtigung des Nutzen-Risiko-Verhältnisses die optimale altersgerechte Dosierung auswählen. Dabei sind sowohl haftungsrechtliche Aspekte als auch die Erstattungsfähigkeit durch die gesetzlichen Krankenkassen zu beachten [26]. Sollte es zu einem Rechtsstreit kommen, stehen die Ärztinnen und Ärzte in der Beweispflicht. Die Verfügbarkeit von Off-Label-Dosierungsempfehlungen mit klarer Referenz zu der besten verfügbaren zugrunde liegenden Evidenz ist daher von großer Bedeutung.

Im stationären Bereich werden mehr Off-Label-Medikamente verordnet als im ambulanten Bereich [3]. Insofern stimmen die Angaben in der Literatur mit den Ergebnissen der Nutzerumfrage überein, dass 74 % der Befragten im stationären Bereich tätig sind, während nur 25 % im ambulanten Bereich beschäftigt sind.

Die meisten Befragten sind vor allem an Dosierungsempfehlungen, aber auch an anderen Informationen wie dem Zulassungsstatus und altersgerechten Präparaten interessiert. Während die Dosierungsempfehlungen die Ergebnisse eines internationalen und zwischen den beteiligten Ländern harmonisierten Prozesses sind, stellen der Zulassungsstatus und die Informationen zu den Präparaten rein nationale, länderspezifische Inhalte dar [9, 18].

Der Wunsch nach der Entwicklung einer App, die einen Offlinezugang zu den Informationen von Kinderformularium.DE ermöglicht, dominierte auch in dieser Umfrage die „Wunschliste“ der Befragten. Voraussetzung hierfür ist zunächst eine stabile Finanzierung für die Erstellung und Pflege der Inhalte der Datenbank. Dann können im nächsten Schritt zusätzliche Funktionalitäten erarbeitet werden.

Während in anderen Ländern eine staatliche Finanzierung längst etabliert ist, wird Kinderformularium.DE genauso wie Embryotox.de, ein ebenfalls längst etabliertes Nachschlagewerk für die Arzneimitteltherapie in der Schwangerschaft und Stillzeit, noch immer projektbezogen finanziert.

### Limitationen

Für die Analyse von Kinderformularium.DE wurden spezifische Altersgruppen berücksichtigt. Die Empfehlungen aus dem Kinderformularium.DE stimmen jedoch nicht immer mit den in dieser Analyse festgelegten Altersgruppen überein. Um eine Überbewertung der Off-Label-Datensätze zu vermeiden, erfolgte eine Einstufung der Empfehlungen als *on*-label, auch wenn diese nur für einen Teil der Altersgruppe galten. Das ermittelte Verhältnis von On-Label- zu Off-Label-Empfehlungen ist daher als konservative Einschätzung zu verstehen.

Bei der Interpretation der Nutzerumfrage ist zu berücksichtigen, dass im Rahmen der Auswertung sowohl die Antworten von Erstnutzenden als auch von häufigen und langjährigen Nutzenden in die Bewertung einbezogen wurden. Die Erfahrungen hinsichtlich der Struktur und Nutzung der Datenbank können jedoch stark variieren. Des Weiteren besteht die Möglichkeit, dass es zu einer gewissen Stichprobenverzerrung gekommen ist. Ehemalige Nutzende, die die Datenbank aus verschiedenen Gründen, wie beispielsweise Unzufriedenheit, nicht mehr nutzen, konnten nicht erreicht und ihre Meinungen somit nicht erfasst werden. Außerdem sind Informationsverzerrungen bei retrospektiven und subjektiven Aussagen nicht auszuschließen. Extrem positive oder negative Positionen prägen sich tendenziell stärker ein.

## Fazit

Kinderformularium.DE ist ein in Deutschland etabliertes Nachschlagewerk für die pädiatrische Arzneimitteltherapie und wird von Ärztinnen und Ärzten, aber auch anderen Berufsgruppen häufig benutzt. Es trägt zur Optimierung der Verfügbarkeit von (Off-Label‑)Dosierungsempfehlungen sowie zum Zugang zu sicheren Medikamenten in der pädiatrischen Versorgung bei.

Die internationale Zusammenarbeit mit dem niederländischen Kinderformularium und weiteren Partnern aus Österreich und Norwegen ermöglicht durch die Bündelung von Ressourcen und Kompetenzen die schnelle Entwicklung und Weiterentwicklung der Plattform vor allem hinsichtlich der Dosierungsempfehlungen. Rein nationale Informationen wie der Zulassungsstatus und die Verfügbarkeit von kindgerechten Präparaten unterstützen die Nutzenden zusätzlich und werden regelhaft genutzt.

Die Ergebnisse der Umfrage unterstreichen die Relevanz von Kinderformularium.DE für die medizinische und pharmazeutische Praxis und belegen ein hohes Maß an Zufriedenheit mit der Datenbank. Da die verfügbare Evidenz für Off-Label-Therapien kontinuierlich zunimmt, muss die Datenbank auch in Zukunft stetig aktualisiert und um zusätzliche evidenzbasierte Dosierungsempfehlungen ergänzt werden, um eine weitere Verbesserung der AMTS in der Pädiatrie zu gewährleisten. Ein Abgleich der tatsächlich eingesetzten Arzneimittel zum Beispiel aus elektronischen Verordnungssystemen mit den Inhalten der Datenbank kann helfen, Kinderformularium.DE weiter zu optimieren und die relevanten Informationen bereitzustellen.

## Supplementary Information

Fragebogen zur Nutzerumfrage Kinderformularium.DE (November 2024–Februar 2025)

## Data Availability

In dieser Arbeit wurde die öffentlich zugängliche Datenbank Kinderformularium.DE ausgewertet. Die Umfragedaten wurden von den Autoren archiviert, die Ergebnisse der Nutzerumfrage wurden in dieser Arbeit dargestellt.
