# Supplementary material for: Evidence-based drug information for children and adolescents in Germany—Are we moving in the right direction?
Source: Bundesgesundheitsblatt Gesundheitsforschung Gesundheitsschutz. 2026 May 8;69(6):635–43. [Article in German] doi: 10.1007/s00103-026-04239-1 (PMC13212619; doi:10.1007/s00103-026-04239-1)
Supplement: Supplementary file 1 — Fragebogen zur Nutzerumfrage Kinderformularium.DE (November 2024–Februar 2025) [file 103_2026_4239_MOESM1_ESM.pdf]

Fragebogen zur Nutzerumfrage Kinderformularium.DE (November 2024 – Februar 2025)

Bitte beantworten Sie folgende Fragen, indem Sie jeweils die zutreffende Antwortoption auswählen (*bei einzelnen Fragen ist eine Mehrfachantwort bzw. Freitextantwort möglich*):

Frage 1: Ich nutze das Kinderformularium.DE heute als...

- ◻ Arzt/Ärztin
- ◻ Pflegefachfrau/-mann
- ◻ Apotheker/-in
- ◻ PTA
- ◻ Studierende/-r
- ◻ Betreuungsperson
- ◻ Sonstiges (bitte angeben*, Freitextantwort*)

Frage 2: In welchem Bereich sind Sie tätig?

- ◻ Klinik
- ◻ Praxis/andere ambulante Einrichtung
- ◻ Krankenhausapotheke
- ◻ Öffentliche Apotheke

Frage 3: Welcher Altersgruppe gehören Sie an?

- ◻ 18-25 Jahre
- ◻ 26-35 Jahre
- ◻ 36-45 Jahre
- ◻ 46-55 Jahre
- ◻ 56-65 Jahre
- ◻ 66-75 Jahre
- ◻ 76 Jahre oder älter

Frage 4: Ich kenne das Kinderformularium.DE durch... *(Mehrfachantwort ist möglich)*

- ◻ Google
- ◻ Fachzeitschriften
- ◻ Kolleginnen und Kollegen
- ◻ Studium, Fort- /Weiterbildungen
- ◻ Soziale Medien
- ◻ Mitteilungen der Berufsgruppe
- ◻ Sonstiges (bitte angeben*, Freitextantwort*)

Frage 5: Wie oft nutzen Sie das Kinderformularium.DE?

- ◻ Mehrmals täglich
- ◻ 1x täglich
- ◻ Mehrmals wöchentlich
- ◻ 1x wöchentlich
- ◻ 1x monatlich
- ◻ Seltener

Frage 6: Wie lange nutzen Sie das Kinderformularium.DE schon?

- ◻ Seit mehreren Jahren
- ◻ Seit einem Jahr
- ◻ Seit Monaten
- ◻ Seit Wochen
- ◻ Seit heute
- ◻ Keine Angabe

Frage 7: Nach welchen Informationen haben Sie gesucht? *(Mehrfachantwort ist möglich)*

- ◻ Zulassungsstatus (on-/off-label)
- ◻ Dosierungsempfehlungen für Kinder
- ◻ Kindgerechte Arzneiformen
- ◻ Informationen zu Rezepturen
- ◻ Informationen zur Pharmakodynamik
- ◻ Kinderspezifische Daten zur Pharmakokinetik
- ◻ Informationen zum Therapeutischen Drug Monitoring
- ◻ Kinderspezifische Informationen zu Dosisanpassungen bei Nierenfunktionsstörungen
- ◻ Kinderspezifische unerwünschte Arzneimittelwirkungen/Warnhinweise/Kontraindikationen
- ◻ Wechselwirkungen
- ◻ Sonstiges (bitte angeben*, Freitextantwort*)

Frage 8: Finden Sie Antworten auf Ihre gesuchten Fragen im Kinderformularium.DE?

- ◻ Immer
- ◻ Oft
- ◻ Selten
- ◻ Nie
- ◻ Für folgende Frage habe ich keine Antwort gefunden: *(zusätzliche Freitextantwort)*

Frage 9: Ich finde die Informationen des Kinderformularium.DE zuverlässig.

- ◻ Stimmt völlig
- ◻ Stimmt eher
- ◻ Teils-teils
- ◻ Stimmt eher nicht
- ◻ Stimmt überhaupt nicht
- ◻ Grund (optional, *zusätzliche Freitextantwort*)

Frage 10: Ich finde die Informationen des Kinderformularium.DE aktuell.

- ◻ Stimmt völlig
- ◻ Stimmt eher
- ◻ Teils-teils
- ◻ Stimmt eher nicht
- ◻ Stimmt überhaupt nicht
- ◻ Grund (optional, *zusätzliche Freitextantwort*)

Frage 11: Ich finde die Informationen des Kinderformularium.DE vollständig.

- ◻ Stimmt völlig
- ◻ Stimmt eher
- ◻ Teils-teils
- ◻ Stimmt eher nicht
- ◻ Stimmt überhaupt nicht
- ◻ Grund (optional, *zusätzliche Freitextantwort*)

Frage 12: Ich finde die Informationen des Kinderformularium.DE verständlich.

- ◻ Stimmt völlig
- ◻ Stimmt eher
- ◻ Teils-teils
- ◻ Stimmt eher nicht
- ◻ Stimmt überhaupt nicht
- ◻ Grund (optional, *zusätzliche Freitextantwort*)

Frage 13: Ich finde die Informationen des Kinderformularium.DE unabhängig.

- ◻ Stimmt völlig
- ◻ Stimmt eher
- ◻ Teils-teils
- ◻ Stimmt eher nicht
- ◻ Stimmt überhaupt nicht
- ◻ Grund (optional, *zusätzliche Freitextantwort*)

Frage 14: Die Website Kinderformularium.DE ist benutzerfreundlich.

- ◻ Stimmt völlig
- ◻ Stimmt eher
- ◻ Teils-teils
- ◻ Stimmt eher nicht
- ◻ Stimmt überhaupt nicht
- ◻ Grund (optional, *zusätzliche Freitextantwort*)

Frage 15: Ich finde es einfach, schnell die Informationen zu finden, die ich suche.

- ◻ Stimmt völlig
- ◻ Stimmt eher
- ◻ Teils-teils
- ◻ Stimmt eher nicht
- ◻ Stimmt überhaupt nicht
- ◻ Grund (optional, *zusätzliche Freitextantwort*)

Frage 16: Übernehmen Sie die empfohlenen Dosierungen?

- ◻ Immer
- ◻ Meistens
- ◻ Manchmal
- ◻ Nie
- ◻ Begründung (optional, *zusätzliche Freitextantwort*)

Frage 17: Nutzen Sie die Zusatzinformationen von Kinderformularium.DE?

- ◻ Regelmäßig
- ◻ Manchmal
- ◻ Nie
- ◻ Zusatzinformationen bisher nicht bekannt

Frage 18: Haben Sie weitere Kommentare und Anregungen? Was können wir besser machen?

- ◻ Kommentare/Anregungen: (*Freitextantwort*)
